# Indoor-related microbe damage induces complement system activation in building users

**DOI:** 10.1177/1753425920966641

**Published:** 2020-12-07

**Authors:** Janne Atosuo, Outi Karhuvaara, Eetu Suominen, Liisa Vilén, Jari Nuutila, Tuula Putus

**Affiliations:** 1 The Laboratory of Immunochemistry, Department of Biochemistry, Faculty of Science and Engineering, University of Turku, Finland; 2 Environmental Medicine and Occupational Health, Department of Clinical Medicine, Faculty of Medicine, University of Turku, Finland

**Keywords:** Complement system, classical pathway, exposure, indoor mold and bacteria, inflammation

## Abstract

In this comparative study, serum complement system antimicrobial activity was measured from 159 serum samples, taken from individuals from microbe-damaged (70 samples) and from reference buildings (89 samples). Antimicrobial activity was assessed using a probe-based bacterial *Escherichia coli*-lux bioluminescence system and comparison was made at a group level between the experimental and reference group. The complement activity was higher in users of microbe-damaged buildings compared with the reference group and the significant (*P* < 0.001) increase in activity was found in the classical reaction pathway. This study strengthens our notion that exposure to indoor-related microbe damage increases the risk for systemic subclinical inflammation and creates a health risk for building users.

## Introduction

The human complement system is composed of about 50 soluble- and cell-bound plasma proteins and glycoproteins, which are essential components of human immune defense mechanisms.^1–3^ The activation of complement by the classical, lectin, or alternative pathway eventually leads to the formation of inflammatory responses that mediate the normal host defenses and tissue homeostasis.^1–4^ The complement system is a highly regulated, self-amplified proteolytic cascade producing various anaphylactic components that promote leucocyte activation and chemotaxis.^1–4^ The formation of the lytic membrane attack complex directly kills and lyses the invading microorganisms, and the enhancement of opsonization and ingestion of the targets during phagocytosis are the direct antimicrobial processes of this system. Moreover, complement participates in Ab responses by augmenting their effects and this complementation is the basis for its name.^1–3^ Finally, the complement system plays a critical role in immune system clearance and in immune complex formation.

Complement functions are a fundamental part of the inflammatory process, and because these functions are able to occur in the absence of infection, the continuous, uncontrollable, and excessive activity constitutes a risk for the host in the form of autoimmune diseases and tissue destruction.

**Figure 1.**
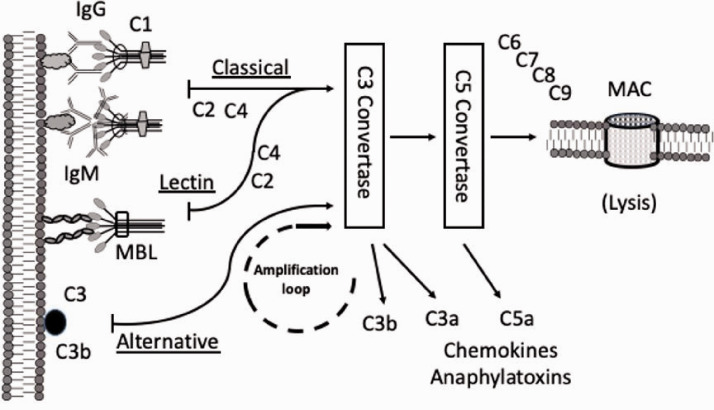
The complement system pathways and effectors. The classical pathway is initiated when the Ag-Ab (IgG and IgM) complex binds with C1 components. The alternative pathway is activated when the C3b fraction (from the C3 tick-over reaction) is committed to the pathogen surface. Man-binding lectin (MBL) attaches to certain carbohydrate residues on the microbe surface activating the lectin pathway. All pathways converge in the C3-convertase state and after the C5-convertase C6, C7, C8, and C9 fragments adhere leading finally to the formation of membrane attack complex (MAC). The complement components C3a and C5a are powerful anaphylatoxins and chemotactic agents that lure leukocytes into the infected tissue and activate them. The released C3b fragment participates in the amplification loop and acts as an opsonin for phagocytic leukocytes.^3^

The complement system includes three separate reaction pathways (Figure 1). The classical activation pathway augments the effects of Abs and is a link connecting the adaptive and innate systems. The main activators are the IgG-class Abs, but also the less specific IgM-pentamer molecules can launch the cascade.^1–3^ Acute phase effectors such as C-reactive protein and amylase are known to directly activate the classical pathway.^5^ The lectin pathway is similar to the classical pathway, but the activation is less specific through the recognition of the mannose residues on the target surface instead of the C1-Ag-Ab complex. The alternative pathway is a part of innate immunity, and it is considered the phylogenetically oldest pathway in mammals.^3,6^ It is initiated by a system called the C3 tick-over that enables rapid conformational changes in the C3 component in the presence of foreign structures.

This study furthers our work from a previous study with the same sample material that showed indoor air-related exposure to microbe-based material (e.g., mold and bacteria) increases spore-specific serum IgG levels in users of microbe-damaged buildings.^7^ Exposure to indoor-related microbe damage, caused by molds and bacteria, elevates the building users’ spore-specific IgG levels at the group level compared with a reference group.^5^ This elevation concerns the total IgG and IgG1, IgG3 ^7^ and IgG2 subclasses (our unpublished data). The same elevation occurs in circulating immune complexes (CIC) concerning the exposed group of building users (our unpublished data). In this study, we analyzed the complement antimicrobial activities from the same serum samples used for the spore-specific IgG^7^ analyses to clarify if this elevated activity would also be present in complement system functions. The comparison between the exposed and reference building users was made at a group level as in previous studies,^7^ and no individual cases were assessed.

We have designed a functional real time-based assay for human complement system activity assessment and previously defined the kinetical parameters of antimicrobial activities concerning the serum classical and alternative pathways. This system is based on the usage of the bacterial probe cell *Escherichia coli*-lux as a target organism.^8–10^ The *E. coli*-lux population emits a constitutive bioluminescence (BL) signal, which correlates to the number of living bacterial cells in the suspension.^8–10^ When an antimicrobial agent is added, the diminishment of the BL signal is directly proportional to the number of killed *E. coli*-lux cells, which has been tested using the plate CFU method.^8,9,11^ This system can differentiate between the classical and alternative pathways, and these parameters were analyzed from the stored serum samples.

## Materials and methods

### Buildings

The microbial damage in the buildings was evaluated, and microbe samples were collected by external experts. Microbe samples were analyzed at the Aerobiology Unit of the University of Turku utilizing the microbe cultivation methods accredited by the Finnish Accreditation Service (FINAS). The moisture-damage microbial panel contained the following indicator microbes: *Aspergillus versicolor, Chaetomium globosum, Fusarium merismoides, Stachybotrys chartarum, Streptomyces albus, Streptomyces halstedii, Trichoderma citrinoviride* and *Tritirachium oryzae*. All three damaged buildings contained microbe growth or severe microbe growth of at least three moisture-damage indicator species (Table 1). No microbial damage was observed in the four reference buildings (Table 1).

**Table 1.** The observed microbial damage of the buildings and the numbers of serum samples collected. Classification of the microbial damages: 0/*** = no microbial growth, */*** = minor microbial growth, **/*** = microbial growth, and ***/*** = severe microbial growth observed.

Microbe-damaged buildings (*n* = 3)	Serum samples (*n* = 70)(F/M = 38/32) Aa = 47.6 ± 3.8Ar = 27 – 76 yrS < 10%
Health care center, Eastern Finland ***/***	26
Apartment building, Southern Finland ***/***	18
Bedroom and kitchen, fire station 1, Southern Finland **/***	26
Non-damaged buildings (*n* = 4)	Serum samples (*n* = 89) (F/M = 53/36) Aa = 41.9 ± 2.15Ar = 20–63 yrS < 10%
Bedroom and kitchen, fire station 2, Southern Finland 0/***	11
Bedroom and kitchen, fire station 3, Southern Finland 0/***	12
Bedroom and kitchen, fire station 4, Southern Finland0/***	7
School, Southern Finland0/***	59

F/M: female/male; Aa: average age: Ar: age range; S: smokers.

### Serum samples

The research material consisted of 70 serum samples from building users, who resided in microbe-damaged buildings, and 89 serum samples from building users from non-damaged buildings (Table 1).

The blood samples were collected from the building users in 8 ml Vacuette serum gel-tubes (Greiner Bio-One, Kremsmünster, Austria). Serum was prepared by centrifugation at 540 *g* for 10 min and then distributed into 2 ml Eppendorf tubes and stored at −80°C. Samples were collected while the individuals worked or lived in the buildings.

### Microbiological analysis

All the buildings were similarly investigated by external experts who also collected the microbe samples. These samples were analyzed at the Aerobiology Unit of the University of Turku utilizing the methods accredited by the FINAS. In cultivation, a dilution culture was used. The total bacterial content and the actinomycete content were determined in THG medium (tryptone-yeast extract -glucose – agar), total spore content of mesophilic fungi in MA2 medium (Malt – agar 2%), and total spore content of xerophilic fungi in DG-18 medium (dichloran-glyserol – agar). The damages and classifications are presented in Table 1.

Refence buildings lacked the damage indicator microbes defined by the analysis service. These indicator species are internationally defined^12^ and this group of microbes contains the fungi and bacteria commonly found from the damaged sites. In our research, these definitions are used to categorize the damaged and non-damaged reference buildings.

### *E. coli*-lux preparation

The recombinant *E. coli* K-12 strain with a plasmid including the modified luciferase gene (pEGFPluxABCDEamp) was used as a bacterial probe cell in complement system activity measurements.^8^ The expression of the whole operon produces the luciferase enzyme complex resulting in *E. coli* cells emitting BL without the addition of substrate.^8^ This strain is designated as *E. coli*-lux.^8^

*E. coli*-lux was cultivated in 100 ml Luria Bertani Broth (LB_amp_) (10 g tryptone (Neogen, Lansing, MI, USA), 5 g of yeast extract (Neogen, Lansing, MI, USA), 5 g NaCl (Sigma-Aldrich, St. Louis, Missouri, USA) and 100 µg/ml ampicillin (Sigma, St. Louis, Missouri, USA, pH 7.4)). The cultivation was incubated in a shaker (250 rpm) at 37°C, until the bacteria suspension was at the end of its logarithmic growth phase at an OD of 0.450, defined by the turbidity measurement at 620 nm (UV-1601 Shimadzu Spectrophotometer, Shimadzu Corp., Tokyo, Japan). Cells were washed and harvested by centrifugation (1500 *g*, 10 min), re-suspended in 10 ml of LB_amp_ containing 25% glycerol, distributed in aliquots, and kept frozen at −80°C. All *E. coli*-lux cultivation media contained ampicillin (100 μg/ml) to maintain the selection pressure.

### Complement activity measurement

The classical complement pathway was measured by adding 50 µl of serum dilution in Hank’s Balanced Salt Solution (HBSS) (Sigma Aldrich) to microtiter plate wells (96-well plate, Greiner One, Düsseldorf, Germany). For the alternative pathway assays, the serum was diluted in HBSS that included 10 mM of the chelating agent ethylene glycol tetraacetic acid (EGTA) and was designated as HBSS_EGTA_. HBSS_EGTA_ removes Ca^2+^ from the reaction leaving only the alternative pathway intact.^8,10^

**Figure 2.**
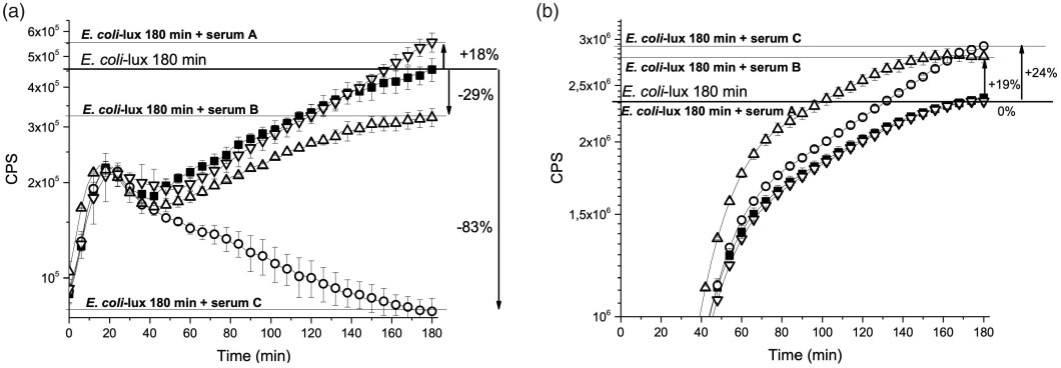
The bioluminescence (BL) kinetics after the 180-min incubation. The black squares represent the well bioluminescence (counts per s, CPS) kinetics without serum (bacteria + Hank’s Balanced Salt Solution (HBSS) or HBSS_EGTA_). Triangles down, triangles up, and circles represent the kinetics of three sample measurements with serum samples, A, B, and C, respectively. (a) Classical pathway, 0.5% serum dilution. After the 180-min incubation, serum A increased the *E. coli*-lux viability to 18%. Sample B diminished the BL signal by 29% (29% killing) and sample C by 83% (83% killing) compared with the bacterial wells. (b) Alternative pathway, 2.0% serum dilution. After the 180-min incubation, serum A had no effect on the *E. coli*-lux viability. Sample B increased the BL signal by 19%, and sample C by 24% compared with the bacterial wells. Values are shown as mean ± SD from three parallel wells.

The reaction was started by adding 50 μl of *E. coli*-lux dilution (1/600 in HBSS or HBSS_EGTA,_ calculatory OD_620nm_ = 0.0075) to the wells containing 50 µl of serum dilution in HBSS or HBSS_EGTA_. The final reaction volume was 100 μl containing approximately 10^5^ (calculated OD_620nm_ = 0.00375) viable *E. coli*-lux cells and 0.5% serum in the classical pathway reactions and 2.0% in alternative pathway assays. These serum dilutions were chosen such that the differences in the antimicrobial activity became visible.^8^ BL was assessed by incubating the microtiter plate in the plate reader luminometer (Hidex Sense Plate Reader, Hidex, Turku, Finland) at 37°C and by measuring the BL signal at 3-min intervals during the 180-min period. Results were shown as the average counts per s per well of three parallel plates counted (Figure 2).

### Data analysis

The percentage of killed *E. coli*-lux cells was counted after 180 min of incubation. The BL signal from the well containing only *E. coli*-lux cells without serum samples was set at 0% of killing. This is presented in Figure 2, showing the kinetic BL signals of *E. coli*-lux suspension incubated with serum samples from three representative building users (A, B, and C).

Raw data were compiled and analyzed using Excel, Version 2016 (Microsoft, Redmond, Washington, USA). Statistical analysis was made with SPSS version 25 (IBM, New York, USA). Due to the nonparametric statistic distribution, the significance of the data was valued using the Mann-Whitney *U* test for calculating the *P* values. Graphs were prepared with Origin, Version 2016 (Microcal, OriginLab, Massachusetts, USA).

## Results

Figures 2a and 2b present the BL kinetics of both classical and alternative pathway, respectively, of three serum samples during the 180-min incubation, and the BL signal of *E. coli*-lux without serum increased during the 180-min incubation. This increase was not because of the proliferation, but due to the BL signal correlating with the increased metabolism, which has been tested in our previous studies using the CFU assessment.^8,9^

**Figure 3.**
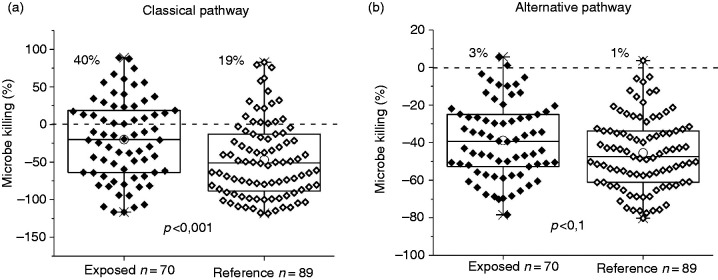
The classical pathway (a) measured by using 0.5% serum dilution and the alternative pathway (b) with 2.0% serum. The microbe killing was counted after 180-min incubation at 37°C. The bioluminescence (BL) signal from the well containing only *E. coli*-lux was set as 0% killing (dashed line) and all the other values were compared with this value. The percentages inside the figures represent the proportion of serum capable of bacterial killing (killing % > 0). The statistic is from 25%, 75%, the line is the median, whiskers are outliers, and circles represent the average crosses at min and max. *P* Values were calculated by using the Mann-Whitney *U* test.

The building users’ sera from the damaged buildings (*n* = 70) manifested significantly higher classical pathway activation (Figure 3a) compared with the serum samples from the reference buildings (*n* = 89). In the group of exposed building users, 40% of samples indicated antimicrobial activity (killing % > 0), whereas in the reference group, this activity was only 19%.

In the alternative pathway, the average complement activity of serum from the exposed building user group was not significantly higher in comparison with the reference building user group (Figure 3b), and only 3% of the exposed serum and 1% of the reference serum killed *E. coli*-lux. As presented in Figure 3b, the antimicrobial activity of the alternative pathway alone was weaker compared with the classical pathway during the 180 min of incubation. EGTA had an effect on the BL kinetics, and in the absence of Ca^2+^, the signal was higher (Figure 2b). Activation of the alternative pathway is Ab independent, and the elevated activities within the group of indoor damage-related microbe-exposed building users (Figure 3b) were not due to the elevated IgG levels.

Serum dilutions were chosen to gain an ideal sensitivity to observe the inter-individual differences in antimicrobial activity (Figures 2 and 3). At low concentrations, some serum samples can cause the enhancement of bacterial viability, as is the case with the serum sample A represented in Figure 2. This same phenomenon is well known in toxicology, because in small doses even highly venomous substances can boost the viability of probe cells.^13^

## Discussion

All the buildings, located in an urban area, were similarly and diligently investigated by external experts who also collected the microbe samples. Samples were collected from inside the building, from damp building materials and from surfaces covered with microbe pulp. Although the external source of the exposure cannot be totally excluded, the indoor subsistence of the microbes was confirmed.

The individual exposure in the building is laborious to determine and depends on the various circumstances such as the time spent in the building, the location inside the building, and susceptibility. However, in this study, the focus was not to compare individuals but the buildings by measuring the complement activity levels of their users.

The hypothesis is that if there is a microbe damage in the building it will more or less, during a course of time, cause the exposure to the building users. This is mostly through the respiratory track and by the dried and dormant microbe material, such as spores and dried mycelium.^7^ Of course, there are interindividual differences between the people residing in these buildings (the possible past exposures, gender, age groups, environmental factors, lifestyle, smokers/non-smokers, diet, etc.) but these are not considered in this manuscript and the group-level comparison is used to reveal the “overall” exposure.

*E. coli*-lux assessment for antimicrobial activity is a convenient real-time based system for analyzing the antimicrobial activities of immune systems, and we have previously used this design to test the antimicrobial functions of human serum. The kinetical parameters of microbial killing by the classical and alternative pathways and the effects of antisera against individual complement components (i.e., anti-C1 and anti-C2) to the microbe killing kinetics have been determined.^8,10^ Moreover, *E. coli*-lux has been used to analyze the antimicrobial activities of serum samples from bats,^14^ rainbow trout^10^ and the antimicrobial activities of insect haemolymph.^15^ We have also used the *E. coli*-lux system to assess antimicrobial mechanisms in neutrophil phagocytosis and to determine lactoperoxidase and myeloperoxidase activities.^11,16,17^

When considering the reasons for the group-level increase in complement system activation against the *E. coli* Ag, we must bear in mind this same test group of exposed building users had increased levels of microbe spore-specific serum IgG,^7,18,19^ and according to our yet unpublished data from the same material, a higher incidence of abnormal CIC levels. Incidentally, the formation of CIC is bound both to the complement activity and the increased level of serum IgG.^20,21^ The elevated complement system activity and especially the enhanced classical pathway suggest subclinical inflammation at the systemic level with elevated rates of complement activation fragments in serum and possibly in affected tissue.^22,23^

All microbe pathogens have a multiple selection of conserved structures called PAMPs,^3^ which are recognized by non-adaptive PRR in serum or on leukocytes, mainly phagocytes.^24,25^ This evolutionary conserved innate system is an essential part of human survival, and it is known to launch the cluster of immune responses stemming from the phagocyte activation and pro-inflammatory cytokine secretion to the activation of the adaptive immune system.

In addition to the activation of the adaptive immune system to produce spore-specific IgGs, prolonged exposure to inhaled microbe-based material (e.g., mold, yeast, and bacteria) may induce the activation of B-1 cells, a subclass of B cells predominantly populating the peritoneal and pleural cavities and capable of generating so-called natural polyclonal IgM Abs.^24,25^

One main route for the exposure to indoor microbe particles is through the respiratory tract, which could enable the activation of B-1 cells in the pleural cavity by PAMPs. Natural polyclonal IgM Abs have the ability to recognize a wide range of PAMPs, some of which can be found also on *E. coli*.^26^ If presented in the serum of exposed building users, these natural M immunoglobulins are able to cross-react with the *E. coli* PAMPs and cause the activation of the classical pathway, offering a feasible explanation to the elevated serum values. The analyses of the *E. coli*-specific IgM levels from the same sample material are underway.

The activation of the alternative pathway is constantly under a rigorous regulation to avoid a futile activation against the host tissue.^27–29^ The first crucial point is the C3 tick-over, a spontaneous process constantly supplying C3b molecules to plasma for “surveillance” purposes. The other is the C3 convertase amplification loop, which could be the source of disorder in the case of an inoperative regulation system. It is not yet known whether the moderately increased activity in the group of exposed building users is caused by the regulatory mechanisms, but the effects of complement regulatory factors on antimicrobial activity are being determined.^30,31^

The activity and the function of the lectin pathway were not analyzed in this study. A reason for this is the difficulty to separate the functional lectin pathway from the classical and alternative pathway.^31^ EGTA inhibits both the classical and lectin pathways, because they are both Ca^2+^ dependent. The inhibition of classical and alternative pathways, in a way that only lectin pathway kinetics would be visible, is not yet possible. This is a setback, because the lectin activation route is related to fungal structures and would be interesting in the case of mold exposure.^5^

For future studies, the analysis of the *E. coli*-specific IgM Abs from the building users’ sera, C-reactive protein (CRP) and serum amylase A, will be investigated. They are both potential classical pathway activators, and increased levels are associated with inflammation and complement activation through the classical pathway.^3,5^ Concurrently with the classical pathway activation, CRP has a dual role acting as an antagonist both to alternative pathway activation and to Membrane attack complex (MAC) formation.^3,5^

## Conclusions

Exposure to indoor microbe damage causes significant complement system activation at a group level compared with the reference material. This elevation was observed in the classical reaction pathway. Further studies will show which factors are the key agonists in complement system activation concerning indoor-related exposure to pathogens.
